# Hypothermic machine perfusion alleviates ischemia-reperfusion injury of intestinal transplantation in pigs

**DOI:** 10.3389/fimmu.2023.1117292

**Published:** 2023-02-28

**Authors:** Wen Hou, Shuang Yang, Jiansen Lu, Yuan Shi, Jing Chen, Decheng Chen, Fei Wang, Lei Liu

**Affiliations:** ^1^ Research Institute of Transplant Medicine, Tianjin First Central Hospital, Nankai University, Tianjin, China; ^2^ National Health Commission’s Key Laboratory for Critical Care Medicine, Tianjin First Central Hospital, Tianjin, China; ^3^ First Central Clinical Institute, Tianjin Medical University, Tianjin, China; ^4^ Tianjin Key Laboratory for Organ Transplantation, Tianjin First Central Hospital, Tianjin, China; ^5^ Organ Transplant Department, Tianjin First Central Hospital, Tianjin, China; ^6^ School of Medicine, Nankai University, Tianjin, China

**Keywords:** hypothermic machine perfusion, intestinal transplantation, ischemia reperfusion (I/R) injury, pig, TLR4/NF κB pathway

## Abstract

**Background:**

Intestinal transplantation (IT) has become an important procedure for the treatment of irreversible intestinal failure. However, IT is extremely vulnerable to ischemia–reperfusion injury (IRI). Due to the limitations of static cold storage (SCS), hypothermic machine perfusion (HMP) is rapidly gaining popularity. In this study, the intestinal HMP system is established and HMP is compared with SCS.

**Methods:**

An intestinal HMP system was built. Ten miniature pigs were randomly divided into the HMP and SCS groups, and their intestines were perfused using the HMP device and SCS, respectively, followed by orthotopic auto-transplantation. Analysis was done on the grafts between the two groups.

**Results:**

Operation success rates of the surgery were 100% in both groups. The 7-day survival rate was 100% in the HMP group, which was significantly higher than that of the SCS group (20%, *P*< 0.05). The pathological results showed that fewer injuries of grafts were in the HMP group. Endotoxin (ET), IL-1, IL-6, IFN-γ and TNF-α levels in the HMP group were significantly lower than in the SCS group (P<0.05), whereas IL-10 levels were significantly higher (P<0.05).The intestinal expression levels of ZO-1 and Occludin were higher in the HMP group compared to the SCS group, whereas Toll-like receptor 4 (TLR4), nuclear factor kappa B (NFκB), and caspase-3 were lower.

**Conclusions:**

In this study, we established a stable intestinal HMP system and demonstrated that HMP could significantly alleviate intestinal IRI and improve the outcome after IT.

## Introduction

Intestinal transplantation (IT) has emerged as the most effective treatment for end-stage intestinal failure (1). Although the overall prognosis of IT has improved significantly when compared to previous transplants, it remains far behind that of other organ transplantation (2–4). The possible reason is that, in addition to the significantly higher risk of rejection and infection than other substantial organ transplants, ischemia–reperfusion injury (IRI) is a major factor influencing IT prognosis (5, 6). Furthermore, of all the abdominal donor organs, the intestinal graft is the most vulnerable, with a significantly shorter cold ischemia time than other organs such as the liver, kidney, pancreas, and lungs (7). Moreover, intestinal IRI is the most severe, causing intestinal mucosal barrier destruction, villi necrosis and shedding, resulting in increased intestinal wall permeability, intestinal microflora heterotopia, and intestinal fluid exudation. It not only impairs intestinal absorption function but also causes systemic inflammatory response syndrome and even sepsis and multi-organ dysfunction.

Several studies, including ischemic preconditioning (8), preservation solution improvement (7), antioxidant and anti-inflammatory treatment (9), and gene therapy (10), have been conducted to prevent and treat IRI, but the vast majority are still in the experimental stage. Thanks to the rapid development of organ preservation technology in recent years (11), it has become a research hotspot in improving donor quality and recipient prognosis. Because of its simplicity and practicability, static cold storage (SCS) technology, which uses UW solution as the main storage solution, has gradually become the standard method for organ preservation since its development in 1987 (12),. SCS was also used in intestinal preservation research (13–15). The principle of SCS technology is that low temperatures inhibit cell metabolism but do not completely stop metabolism. Since SCS technology is no longer capable of meeting the needs of clinical organ preservation, the improvement of small intestine graft preservation technology has become more critical.

Mechanical perfusion technology refers to the continuous perfusion of perfusate to the preserved organs during the organ preservation process. Mechanical perfusion is classified as normothermic machine perfusion (NMP, 32-37°C), subnormothermic machine perfusion (SMP, 20°C) and hypothermic machine perfusion (HMP, 4-6°C) based on the temperature of perfusion system. Recent preclinical studies and clinical trials have demonstrated that NMP preserves the liver better than SCS (16–18). For intestine preservation, NMP was shown to be a viable method for graft preservation (19) and transplantation (20) in porcine transplantation models. HMP technology has been widely used in organ transplantation, particularly kidney transplantation (21–23). This technology offers significant protection against IRI. There are currently only a few studies on the application of HMP for intestine preservation. Yale University researchers attempted to use HMP to preserve the donor intestine *ex vivo*, and pathology revealed that HMP can significantly reduce intestine mucosal injury, indicating promising potential (24, 25). However, HMP has not been applied in IT yet.

In this study, we constructed an intestinal HMP device using a portable compressor refrigerator, a peristaltic pump oxygenator, pressure sensors, temperature sensors, a thrombosis filter, a connecting line, and an organ pool. The protective effect of HMP against intestinal IRI was then tested in porcine orthotopic intestine auto-transplant model.

## Materials and methods

### Animals and material preparation

Bama miniature male or female pigs (10~12 months of age, Bainong Experimental Animal Breeding Technology Co., Ltd, Tianjin, China) were maintained, with 1 animal per cage, in a 12h light/dark cycle condition with access to food and water ad libitum. All experiments were executed based on the Guide for Care and Use of Laboratory Animals (National Institutes of Health, Bethesda, MD, USA) and were approved by the Ethics Committee of Nankai University. Pentobarbital was purchased from Sigma (USA). Atropine was purchased from Jin Yao Pharmaceutical (Tianjin, China). Antibodies for caspase-3, nuclear factor kappa B (NF κB), toll-like receptor 4 (TLR4), Occludin, or ZO-1 were purchased form Abcam (Cambridge, UK). ELISA kits for endotoxin (ET), IL-1β, IL-6, IL-10, IFN-γ, and TNF-α were purchased form TSZ (USA).

The drugs used were as follows: pentobarbital sodium (Sigma, USA), atropine (Tianjin Jin Yao Pharmaceutical, Tianjin, China), heparin sodium (Beijing Huayueyang Biotechnology, Beijing, China), lactated Ringer’s solution (Baxter, Shanghai, China), stroke-physiological saline solution (Shijiazhuang No. 4 Pharmaceutical, Shijiazhuang, China), UW-MPS (Bridge to Life Ltd., USA), and cefuroxime sodium for injection (Esseti Farmaceutici Srl, Italy).

### Experimental design

**Figure 1:**
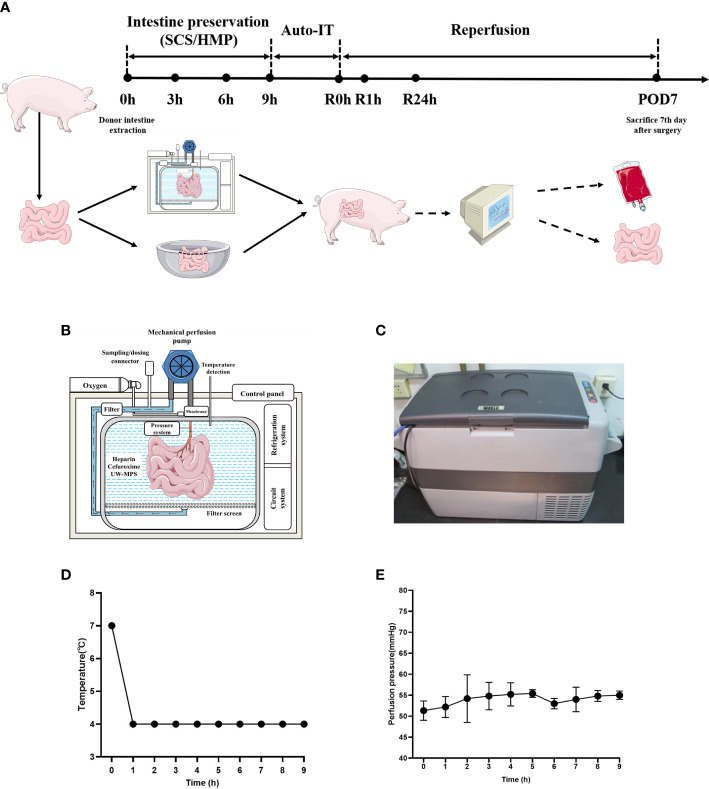
HMP system and study workflow. **(A)** Study workflow. Intestinal grafts were preserved in HMP device (HMP group) and 4°C UW solution (SCS group) for 9 hours and then intestinal auto transplantation was performed. **(B)** Schematic diagram of HMP system. **(C)** Appearance of HMP system. **(D)** Temperature during HMP preservation of small intestine. **(E)** Perfusion pressure during HMP preservation of small intestine (n=5).

10 pigs were randomly divided into 2 groups, SCS (n=5) and HMP (n=5). The pigs were fasted for 36 hours, allowed to drink freely, and given 250 mL 20% mannitol orally before surgery. The donor intestines were preserved for 9 hours with HMP and 9 hours with SCS before orthotropic auto-transplantation was performed on the two groups of experimental pigs (**Figure 1A**).

### Equipment and materials for intestinal HMP devices

The instruments (**Figure 1B**) used were as follows: OEM peristaltic pump (Longer RP5206, series number 701046405, Baoding, China), membrane oxygenator (MAQUET, series number 70058174, Germany), portable compressor refrigerator (MOBICOOLCF-50, Shenzhen, China), temperature monitoring system (custom-made; Shenzhen, China), pressure monitoring system (custom-made; Shenzhen, China), pressure sensor (model DPT-248, SCW Medicath, Shenzhen, China), organ bin (custom-made; Shenzhen, China), heparin cap (Jierui Medical Products, China), three-way valve (Yangsheng Medical Science and Technology, China), thrombosis filter (Tianjin Plastics Research Institute, China), and mechanical perfusion catheter (Tianjin Plastics Research Institute, China).

### Intestinal resection

The pigs were administered with basic anesthesia by intramuscular injection of 3% pentobarbital (25 mg/kg) and atropine (0.03 mg/kg). Then the pigs were tracheally intubated, and hooked up to a ventilator for assisted breathing. Tracheal intubation was performed in IPPV mode, with an oxygen inhalation concentration of 30%–50%, a respiratory rate of 12–16 times/min, a tidal volume of 10–12 mL/kg, and a breathing ratio of 1:2. The anesthetized experimental pigs were placed supine on a constant-temperature operating table, their limbs were fixed, and a blood oxygen saturation probe was attached to the pigs’ tails for continuous ECG monitoring. A longitudinal incision was made through the abdominal midline, exposing the root of the small intestine mesentery and the superior mesenteric artery was separated. To assess the supply intestine, the appropriate jejunal artery branch was carefully separated and blocked. The target length of the intestine was 100–200 cm long. Prior to obtaining the jejunum, 12,500 U of heparin was injected intravenously to heparinize the entire body of the experimental pigs. After 5 minutes, the jejunal artery and vein were blocked and ligated, and 4°C lactated Ringer’s solution (containing 12.5 U/mL of heparin) was perfused through the jejunal artery. The perfusion was maintained until the jejunal vein drained clear fluid, and the mesentery and intestinal wall were pale and bloodless. Following perfusion, the jejunal artery, vein, and corresponding intestine were obtained, and the ends of the intestine tract were ligated after washing out intraluminal intestinal content. The residual intestinal tract of the pigs was anastomosed, and the bleeding was carefully stopped. The abdomen was then closed layer by layer after the automatic retractable hooks were removed. After that anesthesia and muscle relaxants were stopped, and the endotracheal intubation was removed once the experimental pigs were able to breatheon their own. The pigs were then returned to the animal room, which had a constant temperature and humidity. Fluid and analgesic were administered as needed.

### Intestine preservation

In the HMP group, the cannula was placed in the jejunal artery and connected to the HMP device (**Figure 1C**). UW-MPS with heparin sodium (12500U per 1000 mL) and cefuroxime sodium (1.5g per 1000 mL) was used as the perfusate, and the temperature was 4°C (**Figure 1D**). The oxygen flow was set at 0.5–1 L/min, and the oxygen was added into the perfusate by the oxygenator. The perfusion velocity was 60 mL/min and the target pressure was between 50 and 70 mm Hg (**Figure 1E**). In the SCS group, the procured intestine was preserved in the UW storage fluid at 4°C for 9 h. The intestine tissues of both groups were collected at 0h, 1h, 3h, 6h and 9h.

### Intestinal transplantation procedure

The experimental pigs were anesthetized again and tracheal intubation was performed as before. After the anesthesia was stabilized, intestinal auto transplantation was performed. The abdominal cavity was accessed through the original abdominal midline incision, the transplanted intestine was flushed with lactated Ringer’s solution at 4°C through the artery. An end-to-end anastomosis in the artery was performed with a 7-0 Prolene vascular suture, and an end-to-end anastomosis in the vein was performed with a 6-0 Prolene vascular suture. The blood vessels were opened to restore blood flow to the transplanted intestine. The transplanted intestine and the intestinal stump of the experimental pigs were trimmed, and an end-to-end anastomosis of the intestine tract was performed, and the abdomen was closed layer by layer (**Figure S1**). The jugular catheter was secured at the back of the neck. The use of anesthesia and muscle relaxants was discontinued, the tracheal tube was removed after spontaneous breathing recovery of the experimental pigs, and the pigs were returned to the constant-temperature and constant-humidity animal room. Fluid and antalgic were administered as needed. The pigs were sacrificed on the postoperative day 7 (POD7), and exploratory laparotomy was performed. Transplanted intestinal tissue and blood samples were collected and stored for future research.

### Histology and immunohistochemistry assay

The samples for histology were taken from the proximal jejunum of the groups SCS and HMP at 0 h, 1h, 3h, 6h, 9h, R0h and POD 7 (or on the day died). The samples were preserved in a 4% paraformaldehyde solution. The specimens were cut into 5-mm-thick longitudinal sections. Pathology slices were deparaffinized in xylene and rehydrated in an ethanol gradient for hematoxylin and eosin (H&E) staining. An independent pathologist in our hospital performed a blinded histological evaluation. Intestine injuries were assessed using the Park/Chiu scoring system (24) in randomly chosen histological fields at 100× magnification.

Immunohistochemistry was used to examine the expression of Occludin, ZO-1, and TLR4, NF κB, Caspase-3 in two groups at R0h. After deparaffinization and rehydration, antibodies were used at a 1:50 dilution overnight at 4°C. An immunohistochemical color reaction was performed using a 3, 39-diaminobenzidine substrate kit. The slides were counterstained with hematoxylin and dehydrated. The signal was captured and photographed using a microscope at 200× magnification.

### Transmission electron microscopy

The samples for electron microscopy were collected at 1 h, 3 h, 6 h, and 9 h for groups SCS and HMP, respectively, and sectioned into 0.5 mm (3) cubes. Then, store in the electron microscopy solution at 4°C. After washing, fixing, dehydration, embedding, slicing and staining with uranyl acetate and lead citrate, the samples were examined under a transmission electron microscope.

### Elisa assay

Blood samples were collected from the SCS and HMP groups at 0 h, R0 h, R1 h and R24h. The serum was separated from the blood for detecting ET, IL-1β, IL-6, IL-10, IFN γ and TNF-α using an ELISA kit directed by the manufacturer’s instructions.

### Statistical analysis

The statistical software GraphPad Prism 8.0 (GraphPad Software, La Jolla, CA, USA) was used for analysis, and the measurement data were expressed as mean ± standard deviation (SD). T-tests (nonparametric tests) were used to compare the statistical differences between the two groups. Survival analysis was conducted using K-M survival curve comparison. Statistical significance is considered as *P<0.05, **P<0.01.

## Results

### HMP improves survival rate after IT

There were no significant differences in body mass, intestinal length, total operation time (from anesthesia to tracheal intubation removal, excluding 9 h of cold storage time), blood vessel reconstruction time, and intestinal reconstruction time between the SCS and HMP groups (*P*> 0.05; **Table 1**).

**Table 1:** Porcine auto-transplantation surgery of intestine.

Parameters	HMP (n=5)	SCS (n=5)
Mass of pigs (kg)	26.4 ± 12.3	27.3 ± 1.5
Length of donor intestine (cm)	168 ± 22.0	167 ± 14.4
Time of surgery (min)	255 ± 78.3	249 ± 75.4
Time of vascular reconstruction (min)	18.2 ± 2.9	17.8 ± 1.5
Time of intestinal reconstruction (min)	34.6 ± 7.7	33.2 ± 9.6

**Figure 2:**
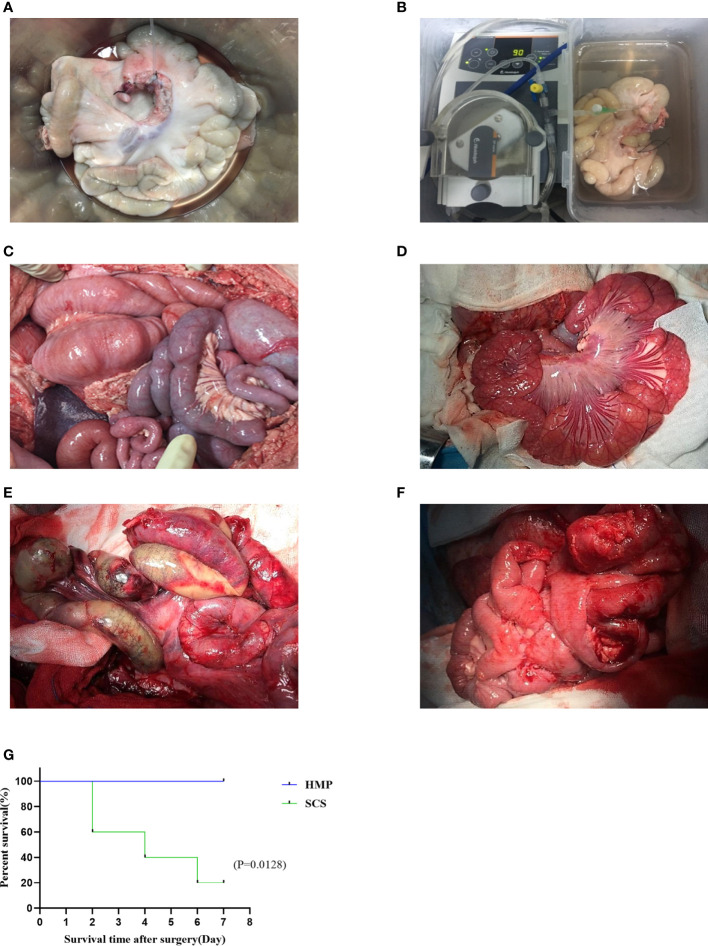
Comparison of transplanted intestine between two groups. The intestines of HMP group **(A, C, E)** and SCS group **(B, D, F)** were observed respectively at 9 h **(A, B)**, R0h **(C, D)**, and POD7 **(E, F)**. **(G)** Survival analysis of two groups of intestine auto transplantation pigs.

After 9 h preservation, the intestinal grafts of the two groups were not significantly different under visual observation (**Figures 2A, B**). During the IT, after the blood flow was restored, the transplanted intestine of the HMP group appeared rosy and had good peristalsis, whereas that of the SCS group appeared dark and swollen with no obvious peristalsis in intestinal (**Figures 2C, D**). A postoperative laparotomy was performed on POD7, and the transplanted intestine of the HMP group appeared rosy and had different degrees of adhesion, however, intestinal necrosis and severe abdominal infection were observed in the SCS group (**Figures 2E, F**).

All the pigs in the two groups survived the operation, and the operative success rate was 100% (10/10). All pigs from the HMP group survived 7 days after the operation, with a survival rate of 100% (5/5). The pigs were defecated 2–3 days after the operation, with no bloody stool. However, only one pig in the SCS group survived 7 days after the operation, with a survival rate of 20% (1/5). Owing to obvious postoperative abdominal distension and intermittent blood stool, two died on POD 2, one died on POD 4, and one died on POD 6. Necropsy revealed intestinal necrosis, intestinal fistula, and severe abdominal infection. There was a significant difference in survival rate after operation between the two groups (*P*< 0.05; **Figure 2G**).

### HMP improves small intestinal blood flow microcirculation

**Figure 3:**
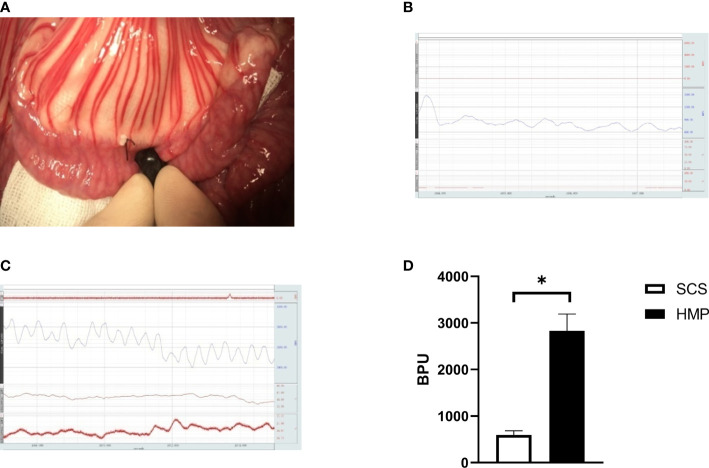
Comparison of microcirculation after blood restore during IT. **(A)** Detection of microcirculation of transplanted intestine by laser Doppler probe. **(B)** The microcirculation of HMP group at R0h, **(C)** The microcirculation of SCS group at R0h, **(D)** Evaluation of the microcirculation (n=5, ^*^P<0.05).

In intestinal auto transplantation, the microcirculation of the transplanted intestine is monitored by a laser Doppler probe after blood flow is restored (**Figure 3A**). The microcirculation of the transplanted intestine was 2,832.60 ± 358.22 BPU in the HMP group and 595.13 ± 90.78 BPU in the SCS group (**Figures 3B, C**), and there was a significant difference between the two groups (**Figure 3D**). The microcirculation of the HMP group was significantly better than that of the SCS group.

### HMP alleviates IRI in pathological examination

**Figure 4:**
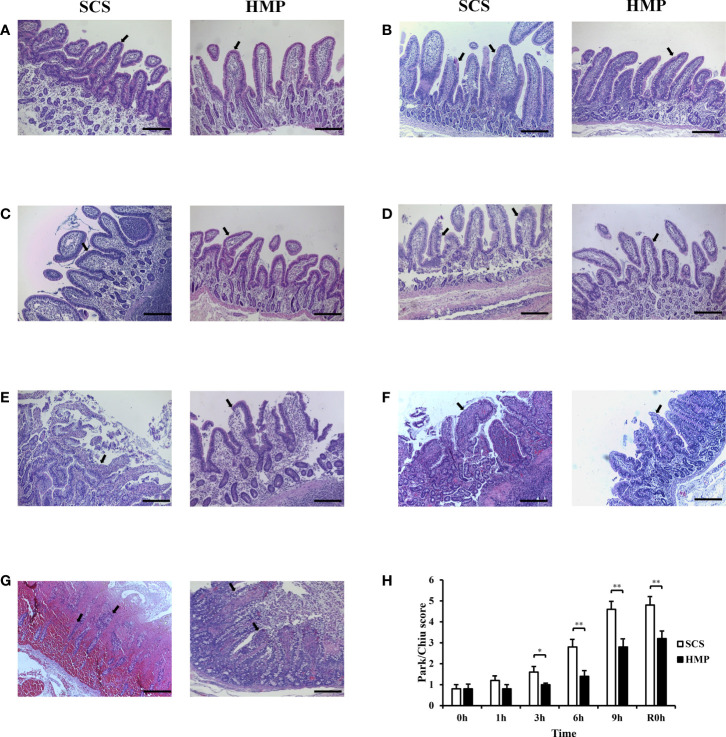
Protective effects of HMP on small intestine preservation. **(A-G)** Representative images H&E staining at 0h **(A)** 1h **(B)** 3h **(C)** 6h **(D)** 9h **(E)** R0h **(F)** POD7 **(G)** of the small intestine. **(H)** Evaluation of the Park/Chiu score. Villi changes and injuries of intestine were indicated by black arrows (original magnification, x100; scale bar, 200μm; n=5; *P<0.05,**P<0.01).

The histopathology of intestinal tissue was examined under the optical microscope (**Figures 4A–G**). It was found that the intestinal mucosal epithelial cells in the HMP and SCS groups were closely connected, neatly arranged and clearly structured at 0 h. Through the preservation, the intestinal mucosal epithelial cells in the HMP group were still intact at 1h, but became mild to moderately edema from 3h to 9h, while the intestinal mucosal epithelial cells in the SCS group were mildly edema at 1h, but became severe edema and were shed and bare villi appeared at 9 h. The Park/Chiu scores of the HMP group were significantly lower than those of the SCS group at 3 h, 6 h, 9 h and R0h (*P*< 0.01; **Figure 4H**), suggesting that HMP has a certain protective effect against intestinal IRI.

**Figure 5:**
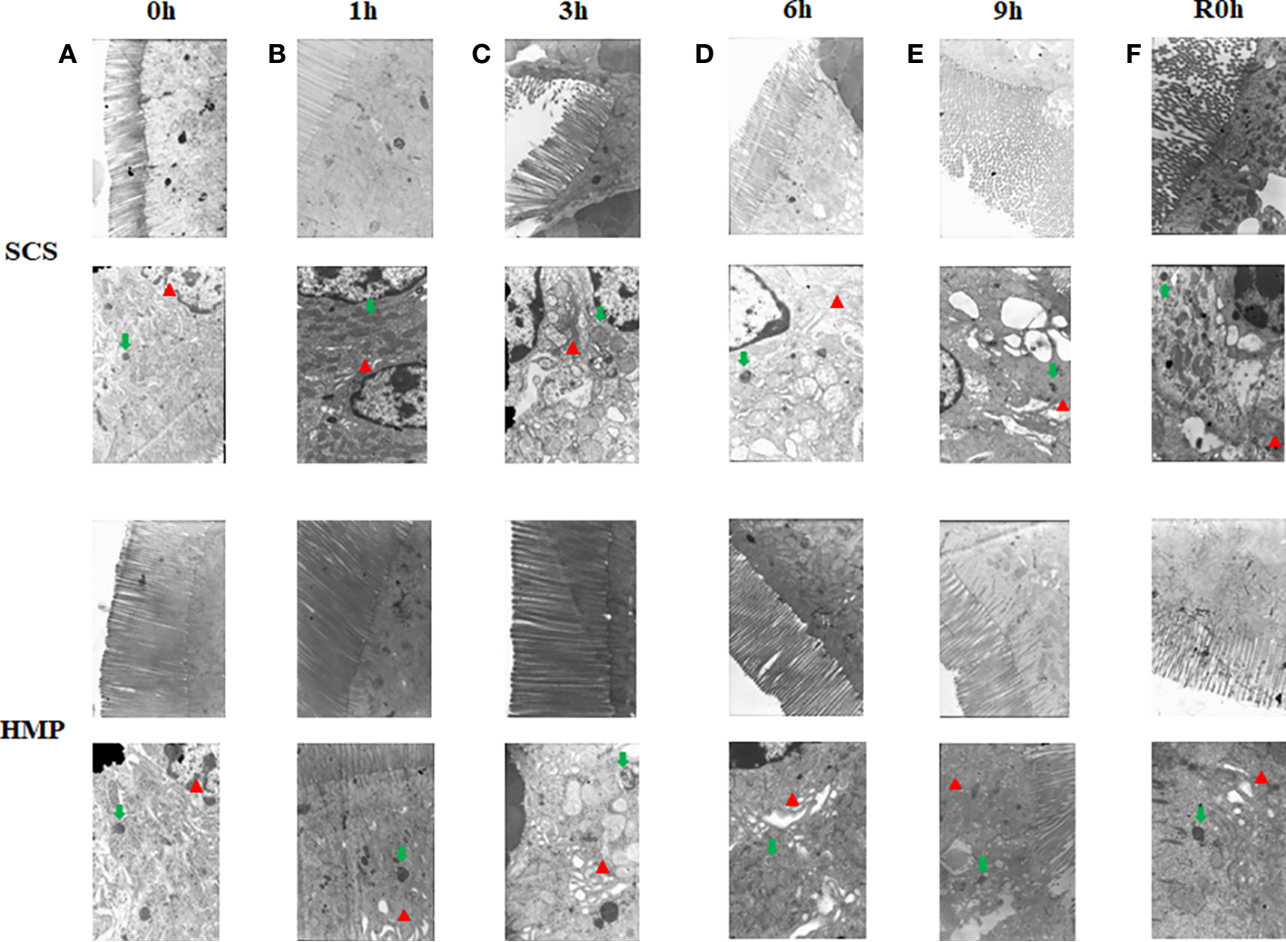
Electron microscopy images of the small intestine at 0h **(A)** 1h **(B)** 3h **(C)** 6h **(D)** 9h **(E)** R0h **(F)**. Endoplasmic reticulum (red arrows) and mitochondria (green arrows) (n=5).

To further evaluate the effect of HMP, ultrastructure of intestine was analyzed by transition electron microscopy. After 1h storage, the microvilli of the HMP group and the SCS group were still neatly arranged, but vacuolar structure was occasionally seen in the cells of SCS group. After 9h storage, a few mitochondria in the HMP group were slightly swollen and the endoplasmic reticulum in a few areas was slightly expanded. In most areas of the SCS group, the microvilli were sparse, and the cells were clearly connected and separated, with vacuoles in the cells. At R0h, the microvilli were sparse in a few areas, the cell connections were basically normal, and the endoplasmic reticulum was slightly expanded in HMP group. However, the microvilli were sparse in most areas of the SCS group, and the cells were connected and separated in some areas, with vacuoles in the cells (**Figures 5A–F**).

### HMP ameliorates intestinal wall permeability and inhibits TLR4/NF-κB pathway

**Figure 6:**
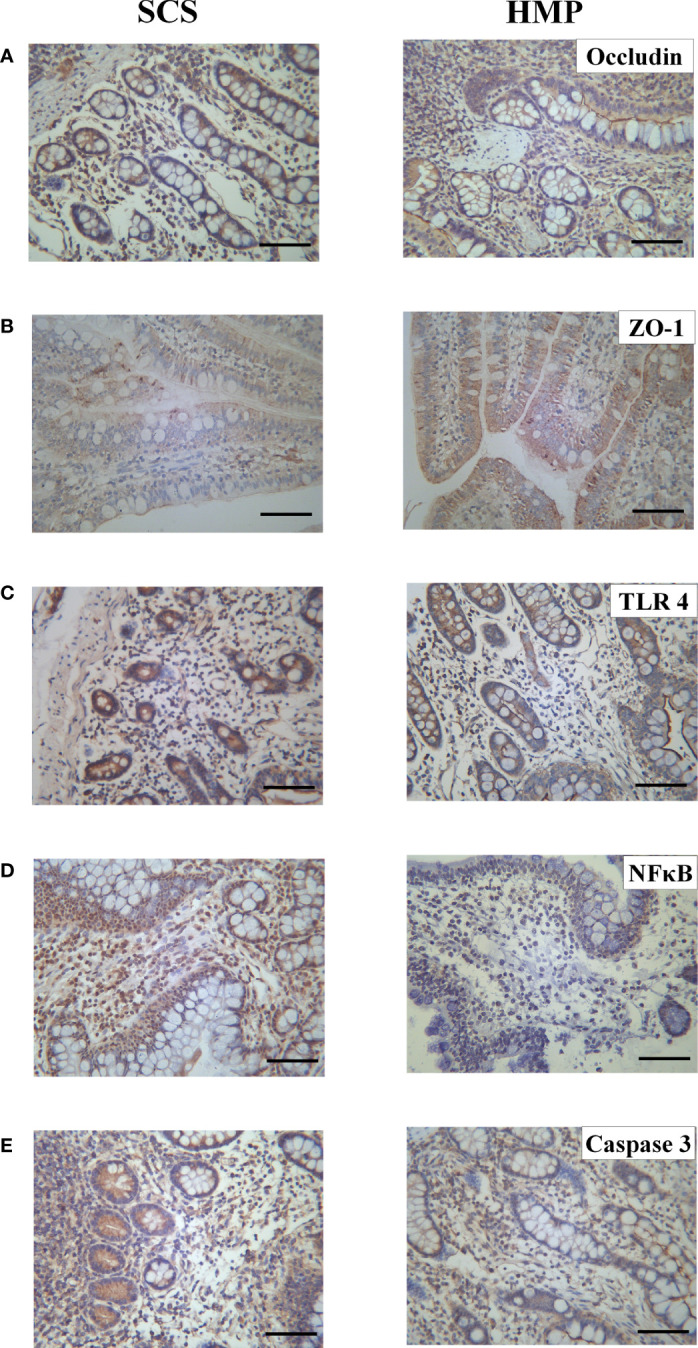
Expression levels of Occludin **(A)** ZO-1 **(B)** TLR4 **(C)** NF кB **(D)** and Caspase 3 **(E)** in intestinal tissues of the two groups by immunohistochemistry. (original magnification, x200; scale bar, 100μm; n=5).

The tight junctions between intestinal cells are an important structural basis for intestinal barrier function and play an important role in regulating intestinal permeability and maintaining the epithelial cell barrier. In order to evaluate the tight junctions of the intestines in SCS and HMP group, we examined expression of the tight junction proteins, such as Occludin and ZO-1 at R0h. Immunohistochemistry analysis showed that compared with the SCS group, the expression of Occludin and ZO-1 were significantly increased by HMP (**Figures 6A, B**), which indicated that the situation of intestinal barrier in HMP was better than that in SCS group.

To evaluate the role of TLR4/NF-κB in HMP on intestinal preservation at the molecular level, we examined expression levels of TLR4, NF-κB and Caspase-3 in the intestine at R0h. TLR4, NF-κB and Caspase-3 expression levels were all lower in HMP group compared to the SCS group, according to immunohistochemistry analysis(**Figures 6C–E**).

### HMP ameliorates inflammation in intestinal IRI

**Figure 7:**
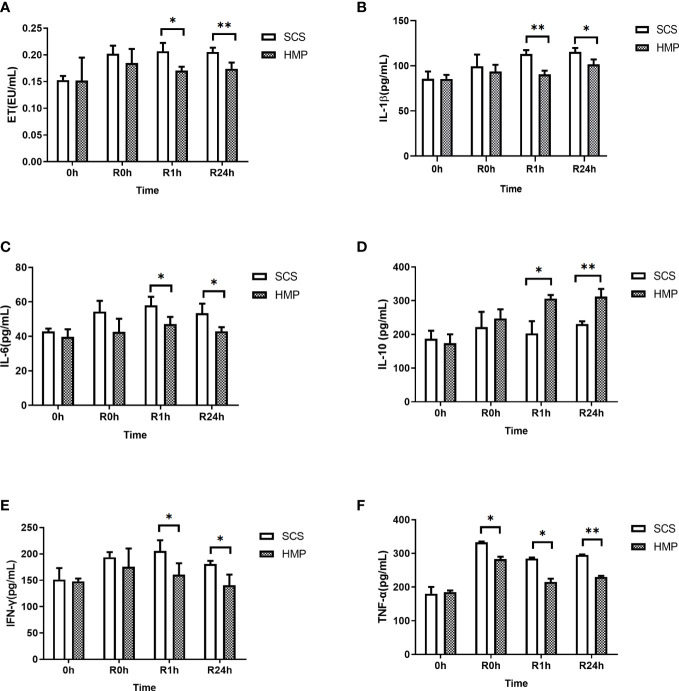
Serum levels of ET **(A)** IL-1β **(B)** IL-6 **(C)** IL-10 **(D)** IFN-γ **(E)** and TNF-α **(F)** at 0h, R0h, R1h, R24h. (n =5; *P<0.05, **P<0.01).

To investigate the role of inflammation in HMP, serum samples from the HMP and SCS groups were analyzed at R0h, R1h and R24h. Serum analysis revealed that the levels of endotoxin (ET), IL-1β, IL-6, IFN-γ, and TNF-α were significantly lower in HMP group compared to the SCS group, while IL-10 level was significantly higher (all *P*< 0.05, **Figures 7A–F**).

## Discussion

In the study of IRI alone, autologous IT with *in vivo* perfusion can meet the requirements. However, if the improvement of perfusion technique is to be discussed, it is still necessary to obtain the transplanted intestine by dissection and perform *ex vivo* perfusion. The majority of existing IT models use the entire small intestine as the donor intestine (26), but the intestinal anatomy of pigs has its particularity. The small intestine is mainly located on the right side of the abdomen, while the colon is located on the left side in front of the small intestine (27) in a spiral arrangement, which is not conducive to the acquisition of small intestine. In the small intestine autograft model established in this study, the intestine supplied by the jejunal artery was selected as the donor intestine, with a length of 120-220cm that was easy to disassemble and moderate in length. This model is simple to operate and has little effect on hemodynamics, making it an ideal animal model for further study of small intestinal graft preservation and IRI.

It has been reported that oxygen consumption at low temperatures (4°C) is about 5-10% of that at normal body temperature. Oxygenated HMP can continuously provide oxygen-containing perfusate through blood vessels, providing nutrition and oxygen at 2-8°C temperature, eliminating metabolic waste, and improving organ quality (28, 29). Many studies have shown that adding oxygen to HMP can provide energy for the heart, liver, kidney and other organs, reduce oxidative stress and repair damage (30–33). In our study, a membrane oxygenator was used to add oxygen to the perfusate during HMP, and it was discovered that intestine IRI was alleviated in the HMP group versus the SCS group. Although oxygen may play an important role in alleviating intestine IRI, more studies are needed to directly compare oxygenated and non-oxygenated preservation techniques in IT, as well as to clarify the significance and mechanism of oxygen in HMP preservation of small intestine grafts.

Cold preservation time is an important factor affecting the prognosis of IT, and the cold ischemia time of IT is currently controlled within 9 h (34). Clinical and animal studies have proved that most small intestine preservation solution can effectively preserve small intestine for 6-8 h (7). In this study, the time limit of 9 hours were used to compare the effects of SCS and HMP.

Researchers from Yale University (24) reported that its self-developed intestinal perfusion unit (IPU) was used for HMP preservation of donor intestine for the first time, and pathological findings indicated that damage to the small intestinal mucosa was significantly reduced. But they did not perform the transplantation. Pathological HE staining suggested that the degree of damage to the small intestinal mucosa preserved by HMP was significantly less than that preserved by SCS, which was consistent with our findings. According to the Park/Chiu score, HMP significantly reduced the degree of intestinal mucosal injury at 3 h after preservation in comparison to the SCS method (*P*< 0.01). This is also confirmed by ultrastructural pathology. After 3 h of cold storage, the villi in most areas of the SCS group were sparse, parts of the cells were separated, and occasional cavitation occurred. After 3 h of cold storage, the cellular structure in the HMP group was still essentially normal. After the cellular structure differences between the two groups became more apparent over time, cell damage in the HMP group was significantly lower than in the SCS group.

Tight junctions are one of the most important structures of the small intestine’s mucosal barrier, consisting primarily of ZO-1, Occludin and other proteins. IRI can lead to damage of tight junctions, destruction of the intestinal mucosal structure, loss of barrier function, and intestinal dysfunction (35, 36). The results of this study showed that the expressions of Occludin and ZO-1 were higher in HMP group than those in SCS group after the preservation process, suggesting that HMP has a protective effect on tight junctions between cells at low temperatures. It is beneficial to maintain the normal structure of the intestinal mucosa barrier.

The intestinal tract is the body’s largest bacterial reservoir. Once the intestinal barrier is breached, the intestinal wall permeability increases, and translocation of microbiota occurs. ET, a lipopolysaccharide component of Gram-negative bacteria’s cell wall, could cross the barrier and enter the intestinal epithelial cell (37). TLR4 is highly expressed in the cell membrane of the gut and is involved in innate immunity. Recognition of specific ligands activates a series of signaling pathways across the membrane, including the NF-κB signaling pathway. NF-κB is an important factor in inducing inflammatory responses and the formation of inflammatory factors (38–40), and it also plays an important role in IRI (41). The caspase family is a key protein in the process of cell apoptosis, and activation and overexpression of this protein will result in cell apoptosis in various tissues (42). Many studies have shown that ET is a powerful activator of TLR4 and can specifically bind to TLR4 to release a large number of inflammatory factors through the TLR4/NF-κB pathway (43–46) and activate caspase-3 to induce apoptosis (36, 47). The results showed that the expression levels of TLR4 and NF-κB and caspase-3 in the HMP group were significantly lower than those in the SCS group during small IT. Meanwhile, we detected a number of cytokines, and the results showed that the expression levels of inflammatory cytokines IL-1β, IL-6, IFN-γ, and TNF-α in the HMP group were significantly lower than those in the SCS group, whereas the expression level of anti-inflammatory cytokine IL-10 was significantly higher than that in the SCS group (*P*< 0.05). These findings suggest that HMP can inhibit the activation of the TLR4/NF-κB pathway and caspase-3, thereby reducing the release of inflammatory factors while increasing the expression of anti-inflammatory factors. This can trigger a systemic inflammatory response, further impairing the function of the intestinal barrier.

## Conclusions

In conclusion, this study is the first to focus on the intestinal IRI of pig intestinal auto-transplantation under the oxygenated HMP graft preservation condition. A stable intestine HMP device was successfully developed, and a stable swine auto-IT model with good stability and repeatability was established. The results indicated that oxygenated HMP may significantly ameliorate intestine IRI and improve the outcome of IT when compared to SCS.

## Data availability statement

The original contributions presented in the study are included in the article/**Supplementary Material**. Further inquiries can be directed to the corresponding author.

## Ethics statement

The animal study was reviewed and approved by Animal Ethics Committee, Nankai University.

## Author contributions

LL and WH designed the study. WH, SY, JC, YS and JL conducted animal and molecular experiments. WH prepared the first draft of the manuscript. DC, FW and LL modified the manuscript. All authors contributed to the article and approved the submitted version.
