# Supplementary material for: Hypothermic machine perfusion alleviates ischemia-reperfusion injury of intestinal transplantation in pigs
Source: Front Immunol. 2023 Feb 28;14:1117292. doi: 10.3389/fimmu.2023.1117292 (PMC10011072; doi:10.3389/fimmu.2023.1117292)
Supplement: Supplementary file 1 [file DataSheet_1.docx]

**Supplementary Information**

In the transplantation surgery, we first anastomosed the jejunal artery by end to end mode, and then the vein. After the blood flow was restored, end to end anastomosis of jejunal tract was performed.


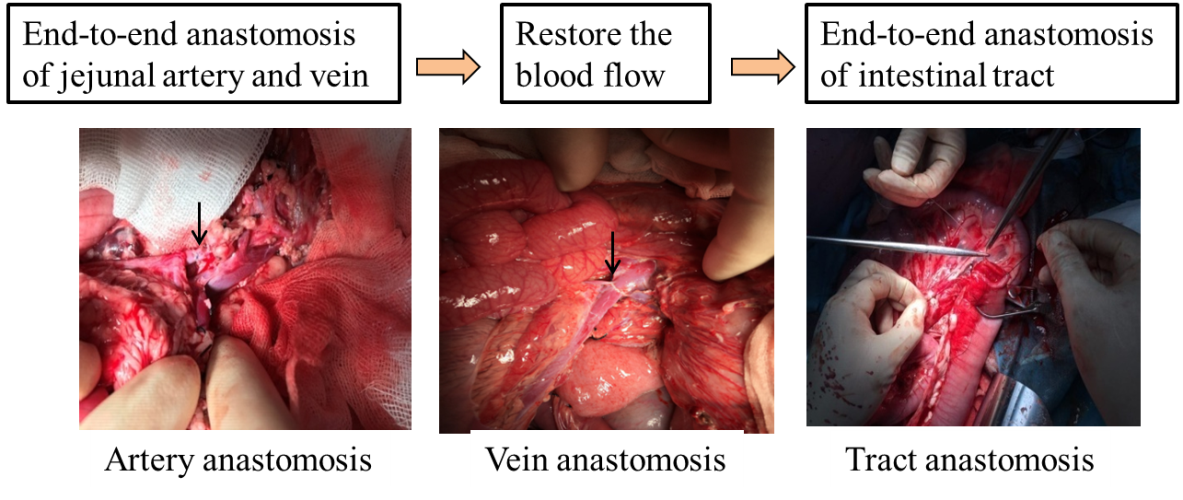


Fig. S1 Jejunal vessels and tract anastomosis
